# What is the Optimal Way to Give Thanks? Comparing the Effects of Gratitude Expressed Privately, One-to-One via Text, or Publicly on Social Media

**DOI:** 10.1007/s42761-022-00150-5

**Published:** 2022-10-11

**Authors:** Lisa C. Walsh, Annie Regan, Jean M. Twenge, Sonja Lyubomirsky

**Affiliations:** 1 University of California, Riverside, CA USA; 2 San Diego State University, San Diego, CA USA

**Keywords:** Gratitude, Well-being, Emotion, Social, Positive activity intervention

## Abstract

Numerous investigations to date have established the benefits of expressing gratitude for improved psychological well-being and interpersonal relationships. Nevertheless, the social dynamics of gratitude remain understudied. Do the effects of gratitude differ when it is expressed privately, communicated directly to the benefactor one-to-one, or shared publicly? We tested this question in a preregistered intervention study. An ethnically and economically diverse sample of undergraduate students (*N* = 916) was randomly assigned to 1 of 4 conditions: (1) write gratitude letters and do not share them (*private gratitude*), (2) share gratitude one-to-one with benefactors via text (*1-to-1 gratitude*), (3) share gratitude publicly on social media (*public gratitude*), or (4) track daily activities (*control*). Participants were asked to complete their assigned activity four times with different people (as applicable) over the course of about a week. Overall, participants assigned to any digital gratitude intervention experienced improvements in state gratitude, positive emotions, negative emotions, elevation, connectedness, support, and loneliness, relative to controls. Relative to all other conditions, participants assigned to text their benefactors showed the biggest boosts in social connectedness and support. Our findings show that easily scalable digital gratitude interventions can advance the well-being of young college students.

**Supplementary Information:**

The online version contains supplementary material available at 10.1007/s42761-022-00150-5.

“No amount of regret changes the past. No amount of anxiety changes the future. But any amount of gratitude changes the present.” —Marc & Angel Chernoff

Most people want to be happy, and gratitude interventions may be an effective path towards this goal (Davis et al., 2015; Diener & Seligman, 2002). Numerous investigations to date have established the benefits of expressing gratitude for improved social relationships, physical health, and psychological well-being (Algoe et al., 2010; Boehm et al., 2011; Bono & McCullough, 2006; Emmons & McCullough, 2003; Fritz et al., 2019; Lambert et al., 2010; Lyubomirsky et al., 2011). However, the social dynamics of gratitude remain understudied. For example, is gratitude more impactful when kept private, shared one-to-one with a benefactor, or shared publicly? Our preregistered, high-powered study aims to fill this gap and advance knowledge about how easily scalable digital gratitude interventions impact well-being.

## Gratitude Defined

The most cited definition of gratitude describes it as a state that requires a person to recognize she has acquired a positive outcome that came from an external source (Emmons & McCullough, 2003). Although most studies conceptualize gratitude as a single, unitary construct, some researchers have proposed an alternative view—that there may be varieties of gratitude (Ahrens & Forbes, 2014; Lambert et al., 2009; Regan et al., 2022). Specifically, gratitude may be classified into two distinct types: (1) *gratitude “for,*” which involves appreciating the positive aspects of one’s life, and (2) *gratitude “to,*” which is prompted by actions performed by benefactors. Recently, the *actor-target-witness framework* proposed that gratitude within dynamic social networks involves distinct roles (actors, targets, and witnesses) and processes (recalling, sharing, receiving, witnessing; Walsh, Regan et al., 2022). Building on this framework, the present study examines gratitude “to” others and compares the effects of actors recalling gratitude privately in their minds (i.e., writing gratitude letters they keep to themselves) vs. directly sharing it with benefactors (either one-to-one via text or publicly on social media).

## Gratitude Interventions

Gratitude interventions are a type of positive activity intervention (PAI). PAIs are simple, low-cost cognitive behavioral strategies that involve mirroring the thoughts and behaviors of naturally happy people (e.g., being thankful, optimistic, or kind) to enhance well-being (Layous & Lyubomirsky, 2014). Furthermore, PAIs can easily be administered by happiness seekers, teachers, researchers, coaches, and/or therapists.

Gratitude interventions usually take one of two primary forms. First, the “counting blessings” (aka “gratitude lists”) intervention that originated in the seminal studies of Emmons and McCullough (2003) instructed participants to write down three to five things they were grateful for (e.g., beautiful nature, their health). Second, the “gratitude visit” (aka “gratitude letters”)—first described by Seligman et al. (2005)—directed participants to write and personally deliver a letter of gratitude to someone who had been especially kind to them (i.e., a benefactor).

Subjective well-being is usually defined as having an affective component that taps positive and negative transient emotions (e.g., happiness, sadness) and a cognitive component (life satisfaction) that assesses overall quality of life (Diener et al., 1999). Hundreds of studies have now shown that gratitude interventions can improve several aspects of subjective well-being (Armenta et al., 2022; Boehm et al., 2011; Emmons & McCullough, 2003; Froh et al., 2009; Lyubomirsky et al., 2011; Seligman et al., 2005). Meta-analytic work also demonstrates that gratitude interventions can effectively boost state gratitude (*d*s = .20 to .46) and well-being (*d*s = .14 to .46), as well as reduce depression and anxiety (*g* = −.29), although some researchers note the effects are small (Cregg & Cheavens, 2021; Davis et al., 2015). A recent critique argued that although gratitude interventions are one of the most frequently recommended happiness strategies to lay people, most of the evidence supporting its effects rely on non-preregistered, low-powered (e.g., *n*s = 20/condition) experiments that predate new open science practices prompted by the replication movement (Folk & Dunn, 2022). Alarmingly, the authors of this critique found only one sufficiently powered, preregistered gratitude study. Such studies are sorely needed to better test the efficacy of gratitude interventions.

Other studies have focused on how gratitude interventions can improve social feelings and relationships. Find-remind-and-bind theory suggests that gratitude serves to strengthen relationships with responsive interaction partners (Algoe, 2012; Algoe et al., 2008). Research supports this theory, as couples prompted to express gratitude to one another often report increases in positive feelings and relationship satisfaction (Algoe et al., 2013; Algoe et al., 2016; Algoe & Zhaoyang, 2015). Additional studies show that gratitude letter interventions can improve general feelings of closeness and social connection (Armenta et al., 2022; Boehm et al., 2011; Layous et al., 2017; Regan et al., 2022; Walsh, Armenta et al., 2022, Walsh, Regan et al., 2022; Wood et al., 2008).

Gratitude letter interventions also impact other outcomes relatively reliably, such as elevation (Armenta et al., 2022; Layous et al., 2017; Regan et al., 2022; Walsh, Armenta et al., 2022, Walsh, Regan et al., 2022), which is characterized by feeling moved, uplifted, and optimistic about humanity (Haidt, 2003; Schnall et al., 2010). Gratitude has also been shown to prompt negative socially oriented feelings, such as guilt, shame, embarrassment, discomfort, and indebtedness (Armenta et al., 2022; Layous et al., 2017; Regan et al., 2022; Walsh, Armenta et al., 2022, Walsh, Regan et al., 2022).

Finally, with the rise of social and digital media (Twenge et al., 2018), some investigators have begun to study the efficacy of digital gratitude interventions. In one study, Sheldon and Yu (2021) assigned undergraduates *(N* = 219; *n* = 49–63/condition) to one of four conditions that directed them to (1) share gratitude with a benefactor face-to-face, (2) share gratitude by video call, (3) share gratitude by text, or (4) control. The study took place in four parts, with each time point spaced 3–5 days apart. All three gratitude conditions boosted well-being, relative to control, but differed little from each other—with the exception that texting gratitude had slightly weaker effects on some outcomes (e.g., loneliness, depression). Another study conducted by Koay et al. (2020) assigned undergraduates (*N* = 33; ~16/condition) to express gratitude on Instagram, and found higher levels of state gratitude, but no effects on life satisfaction, relative to control. Notably, neither study was preregistered and both had small sample sizes. By using a large, high-powered sample and preregistering our hypotheses in advance, we endeavored to test gratitude interventions using open science practices.

## The Current Study

Building on the literature summarized above, the present study aims to examine which ways of expressing gratitude are most beneficial, test the efficacy of digital gratitude interventions, and replicate the effects of previous gratitude studies in a high-powered pre-registered experiment. To these aims, young college students were randomly assigned to one of four conditions: (1) write a gratitude letter and do not share it (*private gratitude*), (2) share gratitude with a benefactor via text (*1-to-1 gratitude*), (3) share gratitude with a benefactor on social media (e.g., Instagram, Facebook; *public gratitude*), or (4) track their daily activities (*control*). Participants were asked to complete their assigned activity four times with different people (as applicable) over the course of about a week. We predicted that participants assigned to any gratitude condition would experience improvements in well-being outcomes, relative to controls. We also expected that sharing gratitude one-to-one might be the most impactful activity because private gratitude does not involve any kind of social interaction (and socializing can increase well-being; Margolis & Lyubomirsky, 2020), while public gratitude may hold disadvantages (e.g., self-censorship, Das & Kramer, 2013). Data were collected February through December 2020.

## Method

### Data Availability

We preregistered our hypotheses on the Open Science Framework (OSF). Preregistration is available on the OSF at https://osf.io/yv9gb. Data, materials, and R code are also available on the OSF at https://osf.io/4bwnf/.

### Preregistered Hypotheses

#### Hypothesis 1

Relative to participants in the *control* condition, those in the gratitude (*private gratitude*, *1-to-1 gratitude*, and *public gratitude*) conditions will experience greater increases in state gratitude, positive emotions, social emotions, life satisfaction, elevation, connectedness, and support, as well as greater decreases in negative emotions and loneliness.

#### Hypothesis 2

Relative to participants assigned to all other conditions, participants in the *1-to-1 gratitude* group will experience the biggest increases in state gratitude, positive emotions, social emotions, life satisfaction, elevation, connectedness, and support, as well as the biggest decreases in negative emotions and loneliness.

### Participants

We recruited 1,105 undergraduate students from a large public university and compensated them with course credit. Eligibility criteria required that they were at least 18 years old, fluent in English, own and use a smartphone, regularly use social media, and have access to an email account they check regularly. We aimed to recruit at least 200 participants per condition (target *N* = 800), but overrecruited to allow for preregistered data exclusions. Following preregistered criteria (and to ensure credibility of responses), 116 participants were excluded for reporting that they did not do the assigned activity, 74 were excluded for putting no effort into the activity, 19 were excluded for completing a survey in less than 2 min, and 13 were excluded for entering the same response more than 15 times in a row. Some participants were excluded for two or more criteria. All exclusions were noted in the preregistration form in either the manipulation check or exclusion criteria sections. Attrition across time points was also relatively low: 4.4% at time 2 (*T*_2_), 6.8% at *T*_3_, and 8.9% at *T*_4_ (the end of the intervention). Dropout across conditions from *T*_1_ to *T*_4_ was relatively comparable: 7.4% for *private gratitude*, 9.3% for *1-to-1 gratitude*, 9.7% for *public gratitude*, and 9.1% for *control*.

The final sample of 916 participants (*M*_age_ = 19.4, *SD* = 2.1) self-reported their genders as 67.7% female, 31.7% male, and 0.7% other (which included “trans male,” “nonbinary,” and “fluid”). Their ethnicities were 42.4% Asian, 33.6% Hispanic, 8.8% White, 3.4% Black, 7.8% more than one race, and 4.0% other. They also came from a range of household incomes: 19.7% reported that their families earned less than $25,000 a year; 23.1% earned $25,000 to $50,000; 26.7% $50,000 to 100,000; 16.6% earned $100,000 to 150,000; 5.2% earned $150,000 to $200,000; and 8.9% earned over $200,000. Most were unpartnered singles (65.6%), but a sizable share were in a romantic relationship of some kind (e.g., married, partnered, cohabitating; 34.4%). Many also worked part-time (27.5%).

### Procedure

**Fig. 1:**
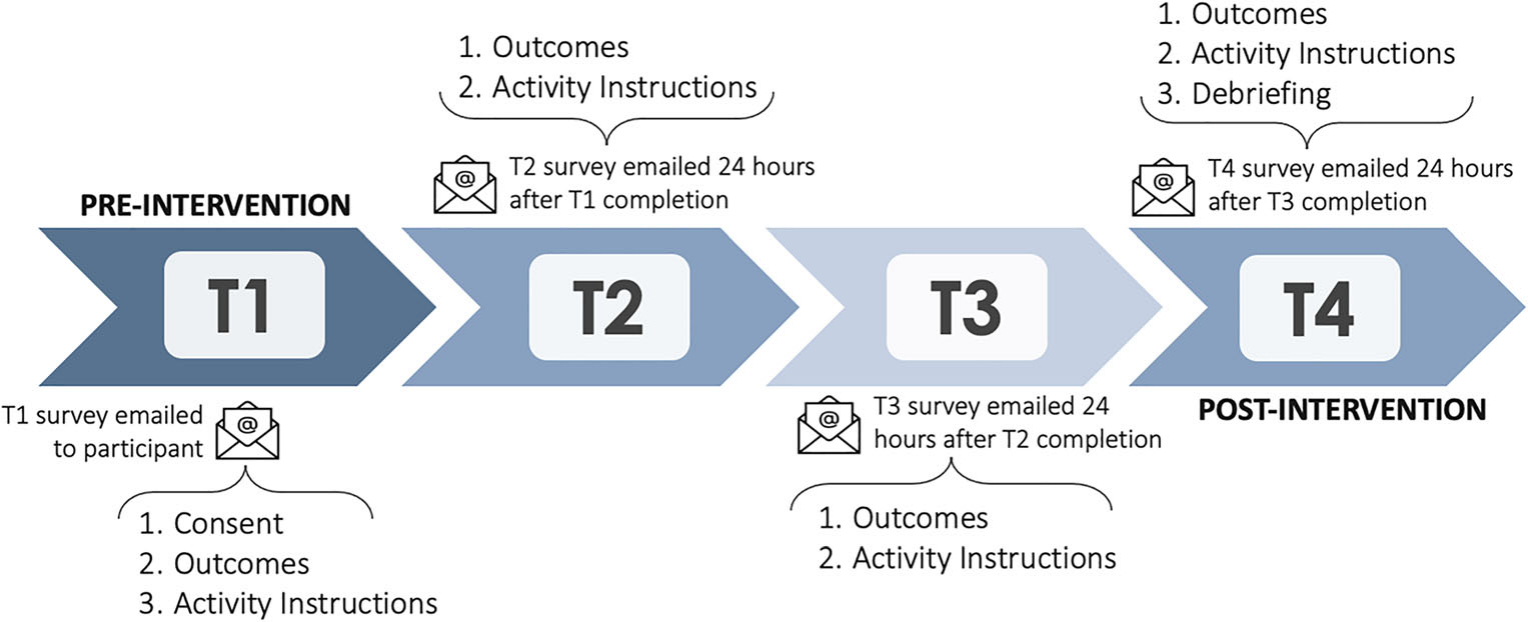
Study timeline. *Note.* The survey for each subsequent time point (e.g., *T*_3_) was sent 24 h after the participant completed the previous time point (e.g., *T*_2_). The study duration was about 1 week (*M* = 4 days; range = 3–15 days). *T*_1_—pre-intervention; *T*_4_—post-intervention

The study took place over four time points that were completed within about a week (see Fig. 1 for study timeline). At the first time point (*T*_1_), participants logged-in to an undergraduate research participation system and were directed to a Qualtrics survey where they consented and completed outcome measures. They were then randomly assigned to one of four conditions. In the *private gratitude* condition, we asked participants to “write a letter of gratitude to someone who has done something for which you are extremely grateful. Please do not share your letter with this person or anyone else.” In the *1-to-1 gratitude* condition, participants were instructed to “use your smartphone to text someone who has done something for which you are extremely grateful, and thank them for their kind act(s).” In the *public gratitude* condition, we asked participants to “use social media to reach out to someone who has done something for which you are extremely grateful, and thank them for their kind act(s).” Finally, participants in the *control* condition were asked to “keep track of all the activities you do” and “keep a brief log.” See Supplemental Materials for full instructions for all conditions. After receiving instructions, they were asked to go off and complete their assigned activity that same day.

Participants were emailed another survey (*T*_2_) a day after completing the *T*_1_ survey. On the second (*T*_2_) survey, we asked participants to report on their experiences and complete additional outcome measures. We then provided similar activity instructions for them to perform again that day. This process repeated for two more time points (*T*_3_ and *T*_4_). Because we aimed to compare three positive interventions with potentially subtle differences, participants were instructed to repeat their assigned activity 3 times over 4 time points (as a way of maximizing effects). We also asked participants in the gratitude conditions to thank a different benefactor at each time point because we wanted the gratitude activity to stay novel and varied, which past research and theory suggests may slow down hedonic adaptation and maximize well-being effects (Fritz et al., 2017; Okabe-Miyamoto et al., 2021; Sheldon & Lyubomirsky, 2012). Studies on hedonic adaptation show that people become happier after positive events (e.g., getting married, writing a gratitude letter), but then revert to their baseline happiness levels over time (Diener et al., 2009). We also expected that participants would find it easier to think of nice things a variety of people had done for them than to think of many nice things a single individual had done for them. Because participants did not receive the next survey (e.g., *T*_3_) until 24 h after they completed the previous survey (e.g., *T*_2_), the study duration (i.e., how many days it took to complete the study) varied slightly by participant, lasting from 3 to 15 days (*M* = 4 days; *SD* = 1.35).

### Measures

#### Gratitude

Gratitude was measured with the three social (i.e., gratitude “to” or person-centered) items from the Gratitude Questionnaire 6-Item (GQ-6; McCullough et al., 2002) and the three social items from the Multi-Component Gratitude Measure (MCGM; Morgan et al., 2017). We did not use the full scales in order to reduce participant fatigue. Example items include “I am grateful to a wide variety of people currently in my life” and “I feel grateful for the people in my life.” Response choices ranged from 1 (*strongly disagree*) to 7 (*strongly agree*). We computed scale reliabilities using Cronbach’s alpha (*T*_1_ *α* = .80; *T*_2_ *α* = .85; *T*_3_ *α* = .85; *T*_4_ *α* = .83).

#### Emotions

Emotions (aka affect) were measured with a modified 17-item Affect-Adjective Scale (AAS; Armenta et al., 2022; Diener & Emmons, 1985; Shin et al., 2021) and split into three types. Positive emotions included items such as “happy,” “pleased,” and “peaceful/serene” (*T*_1_ *α* = .90; *T*_2_ *α* = .92; *T*_3_ *α* = .93; *T*_4_ *α* = .93); negative emotions included items like “angry/hostile,” “depressed/blue,” and “dull/bored” (*T*_1_ *α* = .80; *T*_2_ *α* = .82; *T*_3_ *α* = .81; *T*_4_ *α* = .82); and social emotions included socially oriented negative emotion items like “uncomfortable,” “guilty,” “indebted,” and “ashamed” (*T*_1_ *α* = .80; *T*_2_ *α* = .82; *T*_3_ *α* = .81; *T*_4_ *α* = .82). Participants rated how much they felt each emotion “today,” with response choices ranging from 1 (*not at all*) to 7 (*extremely*).

#### Life Satisfaction

Life satisfaction was measured with 3 items from the Comprehensive Inventory of Thriving (CIT; Su et al., 2014). Example items include “In most ways my life is close to ideal” and “My life is going well” (1=*strongly disagree*; 5=*strongly agree*). *T*_1_ *α* = .83; *T*_2_ *α* = .85; *T*_3_ *α* = .85; *T*_4_ *α* = .88.

#### Elevation

Elevation was assessed with the 6-item elevation questionnaire (Schnall et al., 2010). Example items include feeling “optimistic about humanity” and “a desire to help others,” which participants rated from 1 (*do not feel at all*) to 7 (*feel very strongly*). *T*_1_ *α* = .84; *T*_2_ *α* = .87; *T*_3_ *α* = .89; *T*_4_ *α* = .89.

#### Connectedness

Connectedness (aka relatedness) was measured with 3 items from the Balanced Measure of Psychological Needs (BMPN; Sheldon & Hilpert, 2012) with response choices from 1 (*not at all*) to 5 (*much agreement*). Example items include “I felt a sense of contact with people who care for me, and whom I care for” and “I felt a strong sense of intimacy with the people I spent time with.” *T*_1_ *α* = .84; *T*_2_ *α* = .88; *T*_3_ *α* = .90; *T*_4_ *α* = .90.

#### Support

Support was measured with the 3-item support subscale from the CIT (Su et al., 2014), with response choices from 1 (*strongly disagree*) to 5 (*strongly agree*). Example items include “There are people I can depend on to help me” and “There are people who appreciate me as a person.” *T*_1_ *α* = .86; *T*_2_ *α* = .88; *T*_3_ *α* = .85; *T*_4_ *α* = .83.

#### Loneliness

Finally, loneliness was assessed with the 3-item loneliness subscale of the CIT (Su et al., 2014). Example items were “There is no one I feel close to” and “I often feel left out” (1= *strongly disagree*; 5=*strongly agree*). *T*_1_ *α* = .74; *T*_2_ *α* = .81; *T*_3_ *α* = .82; *T*_4_ *α* = .83.

## Preregistered Analytic Strategy

We also preregistered our analytic plan. First, we created dummy coded pseudovariates to test Hypotheses 1 and 2. For the pseudovariate used to test Hypothesis 1 (Gratitude > Control), the control condition was coded as the reference group. For the pseudovariate used to test Hypothesis 2 (1-to-1 > Others), the other conditions (*private gratitude*, *public gratitude*, and *control*) were coded as the reference group. Next, we examined and plotted the means and standard errors of each condition at each time point to determine what type of model (e.g., linear, quadratic) was most appropriate (see supplemental materials Figure S1). Longitudinal plots showed that change was relatively linear over time, which is consistent with previous gratitude intervention studies modeling linear change (Armenta et al., 2022; Fritz et al., 2019). Thus, we used linear growth model trajectories. We then tested our hypotheses by predicting changes in outcomes (e.g., positive emotions, life satisfaction) via multilevel growth curve modeling, with repeated measures nested within individuals (Bryk & Raudenbush, 1992). Any model with condition predicting slope (i.e., Models 2 and 3) used only one pseudovariate, which meant collapsing across conditions. We first fit an unconditional growth model (Model 1), which was compared to Model 2 (for Hypothesis 1) and Model 3 (for Hypothesis 2). Our preregistration plan also included additional condition comparisons, moderation, mediation, and retrospective change analyses, which are presented in supplemental materials.

## Results

For all results reported below, see Table 1 for multilevel model parameters, standard errors, goodness of fit statistics, and comparisons. Means and standard deviations by condition at each time point, as well as longitudinal plots, are also presented in supplemental materials.

**Table 1:** Multilevel model parameters, standard errors, goodness of fit statistics, and comparisons

		Fixed effects				Random effects							
		Intercept	Time	Condition	Time * Condition	Level 1	Level 2		Goodness of fit			Model comparison	
Outcome	Model	*γ*_00_	*γ*_10_	*γ*_01_	*γ*_11_	*σ*^2^_e_	*σ*^2^	*σ*^2^	AIC	BIC	logLik	∆*χ*^2^	∆df
Gratitude	1. Unconditional Growth	5.94 (0.03)***	0.01 (0.01)	-	-	0.42	0.13	0.68	6557.9	6595.1	−3273.0	-	-
	2. H1: Gratitude > Control	6.02 (0.06)***	−0.06 (0.01)***	−0.11 (0.06)†	0.09 (0.02)***	0.42	0.13	0.68	6531.5	6581.1	−3257.8	30.4***	2
	3. H2: 1-to-1 > Others	5.94 (0.03)***	0.00 (0.01)	−0.02 (0.06)	0.02 (0.02)	0.43	0.13	0.68	6560.0	6609.51	−3272.0	1.95	2
Positive emotions	1. Unconditional Growth	4.06 (0.04)***	−0.02 (0.01)	-	-	0.76	0.22	0.98	10527.8	10565.0	−5257.9	-	-
	2. H1: Gratitude > Control	4.20 (0.09)***	−0.11 (0.03)***	−0.18 (0.10)†	0.13 (0.03)***	0.76	0.21	0.98	10512.8	10562.3	−5248.4	19.0***	2
	3. H2: 1-to-1 > Others	4.07 (0.05)***	−0.02 (0.02)	−0.02 (0.10)	0.02 (0.03)	0.76	0.21	0.98	10531.1	10580.6	−5257.5	0.71	2
Negative emotions	1. Unconditional Growth	2.90 (0.04)***	−0.19 (0.01)***	-	-	0.66	0.18	0.94	9357.8	9395.0	−4672.9	-	-
	2. H1: Gratitude > Control	2.78 (0.08)***	−0.16 (0.02)***	0.16 (0.09)†	−0.05 (0.03)†	0.66	0.18	0.94	9358.0	9407.6	−4671.0	3.79	2
	3. H2: 1-to-1 > Others	2.88 (0.05)***	−0.19(0.013)***	0.09(0.09)	−0.00(0.03)	0.66	0.18	0.94	9360.2	9409.8	−4672.1	1.59	2
Social emotions	1. Unconditional Growth	1.80 (0.03)***	−0.09 (0.01)***	-	-	0.46	0.15	0.83	7012.4	7049.6	−3500.2	-	-
	2. H1: Gratitude > Control	1.79 (0.07)***	−0.08 (0.02)***	0.01 (0.08)	−0.01 (0.02)	0.46	0.15	0.83	7016.4	7065.9	−3500.2	0.08	2
	3. H2: 1-to-1 > Others	1.78 (0.04)***	−0.08 (0.01)***	0.08 (0.08)	−0.02 (0.02)	0.46	0.15	0.83	7015.2	7064.7	−3499.6	1.23	2
Life satisfaction	1. Unconditional Growth	3.23 (0.03)***	0.08 (0.01)***	-	-	0.34	0.11	0.76	5606.7	5642.8	−2796.8	-	-
	2. H1: Gratitude > Control	3.16 (0.06)***	0.06 (0.01)***	0.09 (0.07)	0.04 (0.01)**	0.34	0.11	0.76	5594.4	5644.0	−2789.2	15.26***	2
	3. H2: 1-to-1 > Others	3.21 (0.03)***	0.08 (0.01)***	0.05 (0.06)	−0.01 (0.01)	0.34	0.11	0.76	5608.9	5658.5	−2796.5	0.73	2
Elevation	1. Unconditional Growth	3.86 (0.04)***	0.02 (0.01)	-	-	0.70	0.24	0.96	10190.5	10227.7	−5089.3	-	-
	2. H1: Gratitude > Control	3.93 (0.08)***	−0.09 (0.03) ***	−0.09 (0.10)	0.15 (0.03)***	0.70	0.24	0.96	10164.8	10214.4	−5074.4	29.71***	2
	3. H2: 1-to-1 > Others	3.85 (0.05)***	0.01 (0.02)	0.06 (0.10)	0.03 (0.03)	0.70	0.24	0.96	10191.8	10241.3	−5087.9	2.78	2
Connectedness	1. Unconditional Growth	3.77 (0.03)***	0.08 (0.01)***	-	-	0.45	0.14	0.74	6888.2	6925.3	−3438.1	-	-
	2. H1: Gratitude > Control	3.81 (0.06)***	0.01 (0.02)	−0.07 (0.07)	0.09 (0.02)***	0.45	0.13	0.74	6861.9	6911.4	−3422.9	30.34***	2
	3. H2: 1-to-1 > Others	3.77 (0.04)***	0.06 (0.01)***	−0.03 (0.07)	0.05 (0.02)**	0.45	0.14	0.74	6882.3	6931.9	−3433.2	9.85**	2
Support	1. Unconditional Growth	4.25 (0.02)***	0.02 (0.01)**	-	-	0.32	0.10	0.57	4651.6	4688.7	−2319.8	-	-
	2. H1: Gratitude > Control	4.32 (0.05)***	−0.03 (0.01)**	−0.09 (0.05)†	0.06 (0.01)***	0.32	0.10	0.57	4630.6	4680.2	−2307.3	24.91***	2
	3. H2: 1-to-1 > Others	4.27 (0.03)***	0.01 (0.01)	−0.08 (0.05)	0.03 (0.01)*	0.32	0.10	0.57	4649.8	4699.4	−2316.9	5.69†	2
Loneliness	1. Unconditional Growth	3.00 (0.04)***	−0.21 (0.01)***	-	-	0.45	0.15	1.08	7470.6	7507.7	−3729.3	-	-
	2. H1: Gratitude > Control	2.95 (0.08)***	−0.18 (0.02)***	0.07 (0.09)	−0.04 (0.02)*	0.45	0.15	1.08	7470.0	7519.6	−3727.0	4.55	2
	3. H2: 1-to-1 > Others	2.99 (0.05)***	−0.21 (0.01)***	0.04 (0.09)	−0.02 (0.02)	0.45	0.15	1.08	7472.7	7522.3	−3728.4	1.87	2

*Note*. In Model 1 (unconditional growth), the intercept parameter estimate (*γ*_00_) represents the average outcome at T1 across the sample. In Model 2 (H1: Gratitude > Control) and Model 3 (H2: 1-to-1 > Others), the intercept parameter represents the average outcome for those in the reference group (Control or Others). *AIC*, Akaike information criterion; *BIC*, Bayesian information criterion; *logLik*, log-likelihood. †*p* < .1. *p < .05. **p < .01. ***p< .001

### Hypothesis 1: Gratitude Conditions vs. Control

**Fig. 2:**
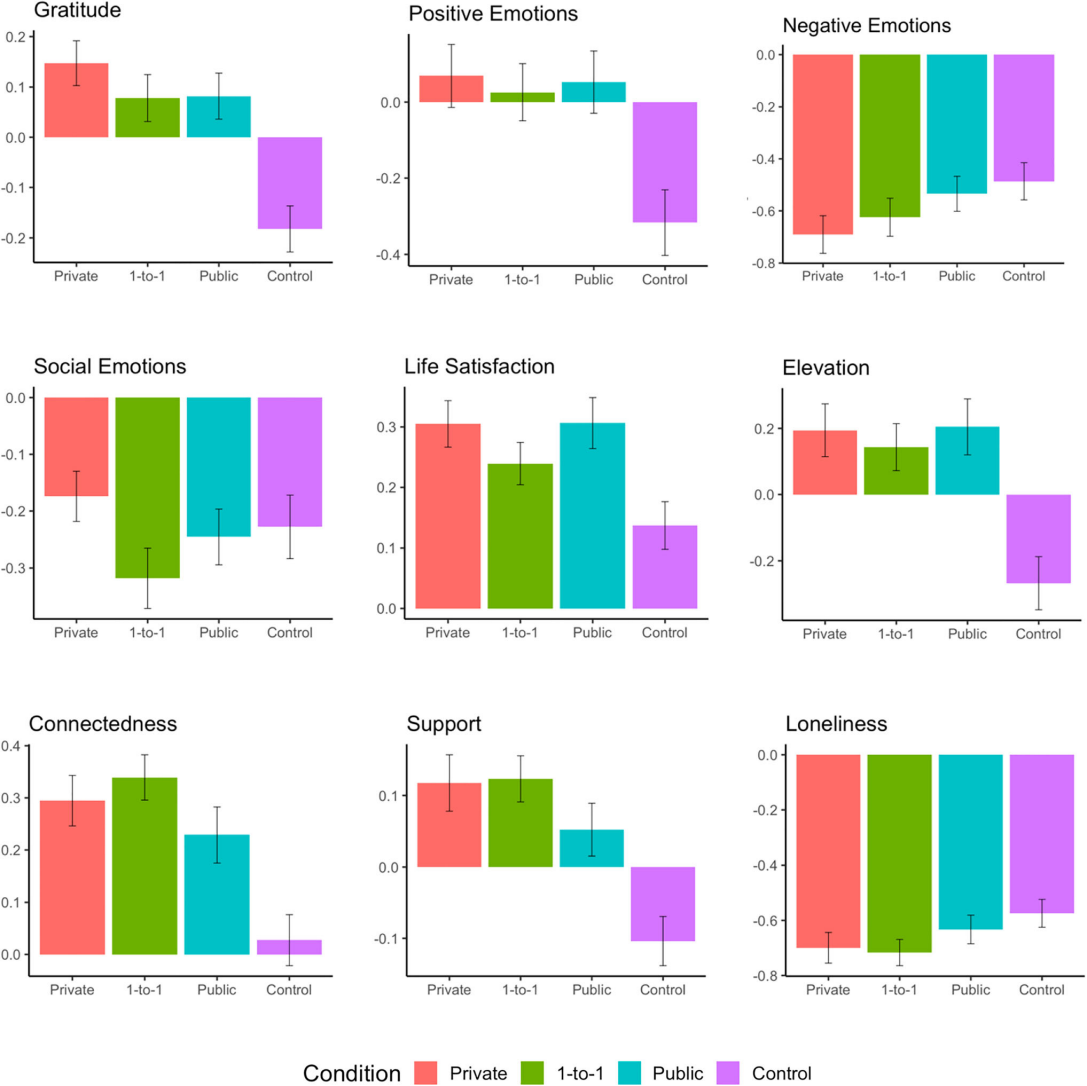
Pre-post difference scores by outcome and condition. *Note.* Pre-post (*T*_4_-*T*_1_) difference scores for all outcomes. Error bars indicate standard errors

Hypothesis 1 was mostly supported. Relative to the control group, participants in the gratitude conditions reported significant increases in gratitude (*γ*_11_ = .09, *p* < .001, partial *d* = .36), positive emotions (*γ*_11_ = .13, *p* < .001, partial *d* = .29), life satisfaction (*γ*_11_ = .04, *p* = .009, partial *d* = .17), elevation (*γ*_11_ = .15, *p* < .001, partial *d* = .35), connectedness (*γ*_11_ = .09, *p* < .001, partial *d* = .33), and support (*γ*_11_ = .06, *p* < .001, partial *d* = .32), as well as significant decreases in loneliness (*γ*_11_ = −.04, *p* = .048, partial *d* = −.13) and marginal decreases in negative emotions (*γ*_11_ = −0.05, *p* = .068, partial *d* = −.12). Interestingly, for some outcomes (e.g., life satisfaction), all of the gratitude conditions improved relative to control, while for other outcomes (e.g., positive emotions, support), the gratitude conditions stayed relatively stable while the control condition actually decreased (see Fig. 2). Social emotions (e.g., shame, indebtedness) did not significantly differ among conditions (*p* = .776).

### Hypothesis 2: Gratitude 1-to-1 vs. All Other Conditions

Hypothesis 2 was mostly unsupported, with two exceptions. Relative to all other conditions (collapsed), the *1-to-1 gratitude* group experienced greater increases in connectedness (*γ*_11_ = .09, *p* <.001, partial *d* = .18) and support (*γ*_11_ = .03, *p* = .017, partial *d* = .16). However, they did not report greater increases in gratitude, positive emotions, social emotions, life satisfaction, and elevation, or greater decreases in negative emotions and loneliness across time (all *p*s > .20).

## Discussion

In an ethnically and economically diverse sample of undergraduate students, we found that those assigned to any type of gratitude intervention (private, 1-to-1, or public) experienced improvements in gratitude, positive emotions, negative emotions, elevation, connectedness, support, and loneliness relative to controls. Additionally, out of all conditions, students assigned to text their benefactors showed the biggest boosts in social connectedness and support. The effect sizes were relatively small (partial *d*s = .12 to .36), but small effects can meaningfully aggregate over time (Funder & Ozer, 2019). Furthermore, because our sample size was large (*N* = 916; *n*s > 210/condition), these effects are likely relatively reliable and replicable. Given that students showed significant improvements within about 4 days, longer digital gratitude interventions may be even more powerful. For example, a recent 4-week gratitude intervention study showed larger effects (*d* = .33) on life satisfaction than did the present study (*d* = .17; Armenta et al., 2022).

Notably, outcomes like life satisfaction, elevation, and connectedness did substantially increase, relative to control, but we saw more of a buffering effect for positive emotions (often considered the hallmark of happiness). In other words, those in the gratitude conditions stayed relatively happy throughout the intervention, but those in the control condition became unhappier. These results are consistent with previous observations that students tend to become less happy as the semester/quarter progresses (e.g., Lyubomirsky et al., 2005); furthermore, our data were collected during the COVID-19 pandemic, which may have been a particularly taxing time in students’ lives (e.g., transitioning to online learning, social isolation).

Although the present study did not examine person-activity fit (i.e., the notion that some interventions may be better for specific individuals than others), past research suggests that allowing people to choose the positive activity that suits them may enhance its well-being benefits (Layous & Lyubomirsky, 2014). Because all three of our interventions “worked,” researchers and practitioners may consider asking people to choose which gratitude activity to try in future endeavors.

Notably, our study is novel for a few key reasons**.** First, we explored whether sharing gratitude 1-to-1 with a benefactor was more beneficial than sharing it publicly or merely recalling it privately. Although some social benefits (connectedness, support) emerged, we found no significant differences in well-being. These social benefits are unsurprising, given that the 1-to-1 condition was the only one that required a personal social interaction (Epley & Schroeder, 2014). Second, our study is one of just a handful to test the effectiveness of digital gratitude interventions (Koay et al., 2020; Sheldon & Yu, 2021). Third, our easy to administer digital gratitude interventions provide a potentially useful template for organizations to use at scale to improve people’s lives. Finally, our large sample size study adds another much-needed, high-powered, preregistered test of the effects of gratitude on well-being (Folk & Dunn, 2022).

### Limitations and Future Directions

Nevertheless, our study has several limitations that may seed future work. Although we endeavored to make condition instructions as parallel as possible, our gratitude interventions may have differed in ways that complicate interpretations, including different levels of participant labor and burden (e.g., writing private letters vs. communicating with benefactors), digital medium (texting vs. social media), and target/benefactor (romantic partners vs. parents vs. friends). For example, writing private letters of indiscriminate length may have required more time and effort than sharing gratitude on social media (or vice versa). Additionally, the assigned digital medium may have influenced whom participants chose as targets. For instance, those sharing gratitude via social media may have been less likely to thank their grandparents, who may not have or use social media. In sum, in testing whether different types of digital gratitude interventions differentially impact well-being, it is unavoidable to create conditions that differ in more than one factor, because digital behaviors themselves differ in more than one factor. Nonetheless, future investigators may seek creative ways to address these issues.

We also gauged participants’ adherence to their assigned intervention instructions with self-report items (e.g., “Yesterday, did you write a letter of gratitude to someone?” [Yes/No]), which may be a less reliable approach than using other more objective criteria. Notably, other prominent positive activity interventions (e.g., acts of kindness) also rely on self-reported adherence items (e.g., Dunn et al., 2008; Ko et al., 2021; Rowland & Curry, 2019). Future studies could collect screenshots of text messages and social media posts as relatively more objective adherence indicators.

Although a strength of our sample was its high ethnic and economic diversity, our participants were more ethnically diverse (42.4% Asian, 33.6% Hispanic, 8.8% White) than samples matched to U.S. census data (6.3% Asian, 18.9% Hispanic, 75.8% White; U.S. Census Bureau, 2021). Additionally, we recruited college students (another oversampled group), so our results may not generalize to the general U.S. population (Brewer & Crano, 2000). Our participants also came from a single, oversampled individualist nation: the USA. Past research shows that gratitude interventions may produce no benefits or even backfire in collectivist cultures (Layous et al., 2013), so large-scale digital gratitude interventions implemented in countries like Japan or Korea may not be as effective. We also cannot speak to the durability of effects presented here because the study took place over about a week and did not include follow-up assessments.

## Conclusion

Overall, the present study shows that digital gratitude interventions helped meaningfully improve students’ well-being—making students feel happier and more satisfied with their lives, as well as more socially connected and less lonely. Furthermore, all three types of gratitude activities (whether they involved writing private gratitude letters, texting benefactors one-on-one, or sharing thanks publicly on social media) benefited participants similarly. However, those assigned to text their benefactors directly reported the biggest boosts in feelings of social connection and support. By examining how digital gratitude interventions do (or do not) affect well-being, we hope this study informs researchers and practitioners about how to cultivate and customize future well-being interventions. Namely, future studies could expand on our approach to determine whether such interventions are similarly beneficial in school districts, companies, governmental organizations, and health care settings. Because digital gratitude interventions can be easily implemented online and feasibly delivered to thousands of individuals, they present a potentially useful tool for both researchers and practitioners.

## Supplementary Information

ESM 1 (DOCX 440 kb)

## Additional Information

### Data Availability

Preregistration is available on the OSF at https://osf.io/yv9gb. Data, materials, and R code are also available on the OSF at https://osf.io/4bwnf/.
