# Supplementary material for: What is the Optimal Way to Give Thanks? Comparing the Effects of Gratitude Expressed Privately, One-to-One via Text, or Publicly on Social Media
Source: Affect Sci. 2022 Oct 11;4(1):82–91. doi: 10.1007/s42761-022-00150-5 (PMC9551243; doi:10.1007/s42761-022-00150-5)
Supplement: Supplementary file 1 — (DOCX 440 kb) [file 42761_2022_150_MOESM1_ESM.docx]

**SUPPLEMENTAL MATERIALS**

Title: What is the Optimal Way to Give Thanks? Comparing the Effects of Gratitude Expressed Privately, One-to-One via Text, or Publicly on Social Media

Authors: Lisa C. Walsh, Annie Regan, Jean M. Twenge, and Sonja Lyubomirsky

**Additional Preregistered Analyses**

**Additional Condition Comparisons**

In line with our preregistration, we tested additional group differences by comparing conditions in an exploratory, pairwise fashion. Because change was relatively linear across time points and conditions (see Figure S1 for longitudinal plots by outcome and Table S1 for means and standard deviations by condition and time point), we ran regressed change models for Hypotheses 1 and 2 predicting T_4_ scores from hypothesized condition dummy coded pseudovariates, controlling for T_1_ scores (see Table S2). These regressed change models resulted in partial *d*s that were highly similar (and often identical) to the multilevel model partial *d*s (see Table S3). Thus, for simplicity, we tested additional condition comparisons with regressed change models (see Table S4). We summarize the results for each of the additional condition comparison regressed change models below.

To further test whether gratitude interventions that involved social interactions (i.e., social gratitude) were more impactful than those that did not (i.e., nonsocial gratitude), we omitted the control condition and used a dummy coded pseudovariate in which the *Gratitude 1-to-1* and the *Gratitude Public* conditions received a “1” and the *Gratitude Private* group was coded as the reference group (receiving a “0”). Participants that shared their gratitude socially (either one-to-one with a benefactor or publicly on social media) did not experience greater improvements in any outcomes than those who wrote a private gratitude letter (see Table S4 Social vs. Nonsocial).

Given that Hypothesis 2 tested whether participants in the *Gratitude 1-to-1* group benefitted more than all other conditions, we ran two additional separate comparisons that tested whether the *Gratitude Private* or *Gratitude Public* groups benefited more than all others. Relative to the other groups, the *Gratitude Private* condition experienced greater increases in state gratitude (*b* = .13, *p* = .013, partial *d* = .17), but there were no significant differences on other outcomes (see Table S4 Private vs. Others). Relative to the other groups, the *Gratitude Public* condition experienced greater increases in life satisfaction (*b* = .10, *p* = .025, partial *d* = .15) and elevation (*b* = .20, *p* = .033, partial *d* = .14), but there were no significant differences on other outcomes (see Table S4 Public vs. Others). Overall, it appears that those in the *Gratitude Private* group experienced the greatest improvements in state gratitude; those in the *Gratitude 1-to-1* group experienced the greatest improvements in connectedness and support, while those in the *Gratitude Public* group experienced the greatest improvements in life satisfaction and elevation.

Finally, we compared each gratitude condition separately to the control condition (with the control coded as the reference group). Relative to control, the *Gratitude Private* condition experienced greater improvements in state gratitude (*b* = .30, *p* < .001, partial *d* = .46), positive emotions (*b* = .35, *p* = .002, partial *d* = .29), life satisfaction (*b* = .18, *p* = .001, partial *d* = .32), elevation (*b* = .47, *p* < .001, partial *d* = .40), connectedness (*b* = .25, *p* < .001, partial *d* = .37), support (*b* = .19, *p* < .001, partial *d* = .38), and loneliness (*b* = -.11, *p* < .001, partial *d* = -.16); however, they did not experience improvements in negative emotions or social emotions (see Table S4 Private vs. Control).

Relative to control, the *Gratitude 1-to-1* condition experienced greater improvements in state gratitude (*b* = .25, *p* < .001, partial *d* = .38), positive emotions (*b* = .33, *p* = .003, partial *d* = .28), life satisfaction (*b* = .13, *p* = .013, partial *d* = .23), elevation (*b* = .44, *p* < .001, partial *d* = .39), connectedness (*b* = .33, *p* < .001, partial *d* = .52), support (*b* = .21, *p* < .001, partial *d* = .45), and loneliness (*b* = -.14, *p* = .02, partial *d* = -.22); however, they did not experiences improvements in negative emotions or social emotions (see Table S4 1-to-1 vs. Control).

Relative to control, the *Gratitude Public* condition experienced greater improvements in state gratitude (*b* = .28, *p* < .001, partial *d* = .43), positive emotions (*b* = .39, *p* = .001, partial *d* = .32), life satisfaction (*b* = .21, *p* < .001, partial *d* = .36), elevation (*b* = .50, *p* < .001, partial *d* = .42), connectedness (*b* = .25, *p* < .001, partial *d* = .36), and support (*b* = .17, *p* < .001, partial *d* = .37; however, they did not experiences improvements in negative emotions, social emotions, or loneliness (see Table S4 Public vs. Control).

In sum, all gratitude groups (*Private*, *1-to-1*, and *Public*) provided similar benefits relative to control, and the effect sizes for each outcome were relatively comparable—further backing up our findings from Hypothesis 1 that suggest any gratitude intervention improved participant outcomes.

**Figure S1**

*Longitudinal Plots by Outcome and Condition*


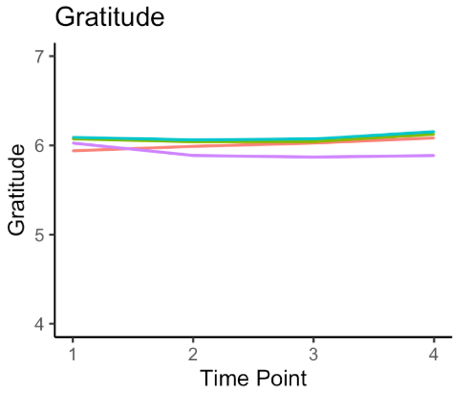

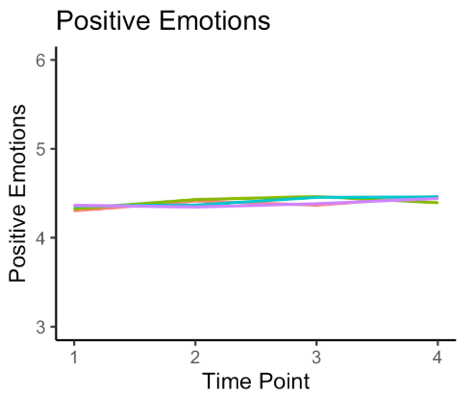

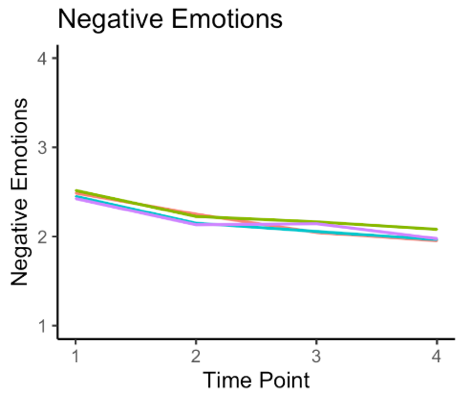


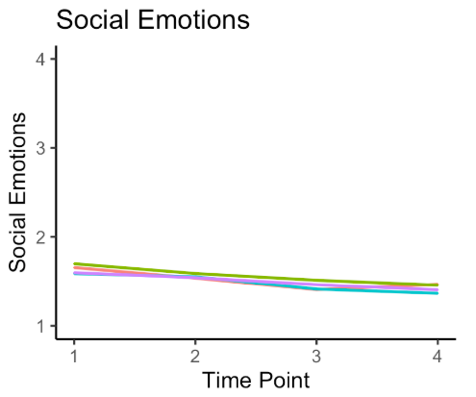

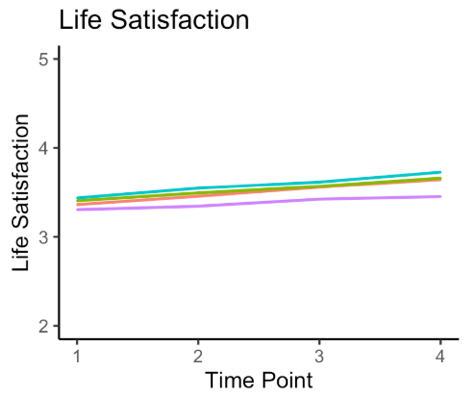

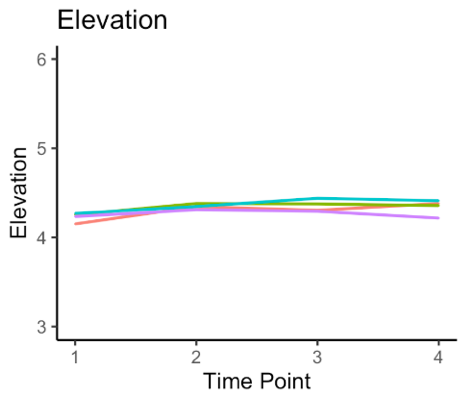


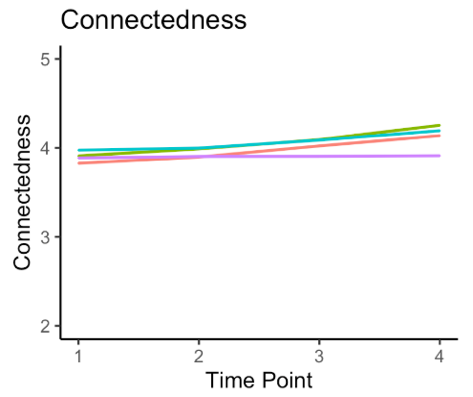

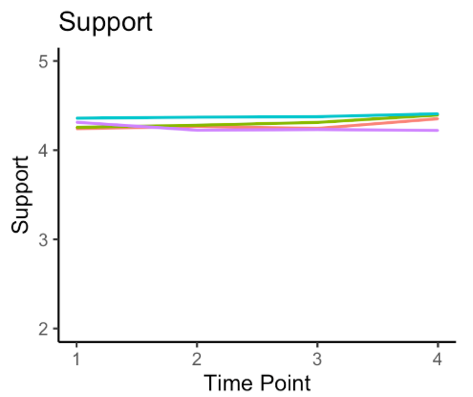

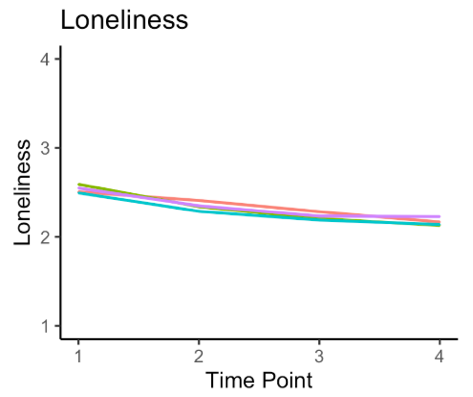


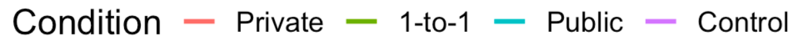


*Note.* Longitudinal plots show that change was relatively linear across time for all outcomes. Standard error bars not included to ease interpretability.

## Table S1

*Means and Standard Deviations by Condition and Time Point*

|  | *Gratitude*  *Private* | | *Gratitude*  *1-to-1* | | *Gratitude*  *Public* | | *Control* | |
| --- | --- | --- | --- | --- | --- | --- | --- | --- |
|  | *M* | *SD* | *M* | *SD* | *M* | *SD* | *M* | *SD* |
| T_1_ Gratitude | 5.89 | .79 | 5.97 | .84 | 6.08 | .70 | 6.01 | .72 |
| T_2_ Gratitude | 5.90 | .84 | 5.96 | .84 | 6.00 | .83 | 5.85 | .81 |
| T_3_ Gratitude | 5.92 | .87 | 5.99 | .89 | 6.02 | .84 | 5.83 | .85 |
| T_4_ Gratitude | 6.06 | .76 | 6.07 | .86 | 6.14 | .75 | 5.83 | .88 |
| T_1_ Positive Emotions | 3.93 | 1.21 | 3.99 | 1.26 | 4.12 | 1.22 | 4.04 | 1.19 |
| T_2_ Positive Emotions | 3.97 | 1.33 | 4.15 | 1.27 | 4.14 | 1.33 | 4.04 | 1.31 |
| T_3_ Positive Emotions | 4.06 | 1.40 | 4.04 | 1.48 | 4.13 | 1.32 | 3.83 | 1.38 |
| T_4_ Positive Emotions | 4.01 | 1.42 | 4.02 | 1.44 | 4.14 | 1.37 | 3.72 | 1.47 |
| T_1_ Negative Emotions | 2.82 | 1.08 | 2.90 | 1.14 | 2.70 | 1.11 | 2.69 | 1.12 |
| T_2_ Negative Emotions | 2.49 | 1.09 | 2.43 | 1.06 | 2.48 | 1.15 | 2.39 | 1.07 |
| T_3_ Negative Emotions | 2.23 | 1.02 | 2.35 | 1.08 | 2.26 | 1.09 | 2.27 | .99 |
| T_4_ Negative Emotions | 2.11 | .99 | 2.28 | 1.11 | 2.16 | 1.05 | 2.19 | 1.11 |
| T_1_ Social Emotions | 1.72 | .85 | 1.80 | .93 | 1.69 | .92 | 1.73 | .96 |
| T_2_ Social Emotions | 1.61 | .82 | 1.62 | .75 | 1.63 | .90 | 1.62 | .86 |
| T_3_ Social Emotions | 1.51 | .84 | 1.55 | .78 | 1.46 | .73 | 1.51 | .75 |
| T_4_ Social Emotions | 1.51 | .74 | 1.50 | .68 | 1.42 | .74 | 1.49 | .85 |
| T_1_ Life Satisfaction | 3.26 | .82 | 3.34 | .83 | 3.41 | .82 | 3.21 | .84 |
| T_2_ Life Satisfaction | 3.39 | .85 | 3.43 | .84 | 3.53 | .78 | 3.25 | .81 |
| T_3_ Life Satisfaction | 3.50 | .79 | 3.49 | .86 | 3.59 | .80 | 3.37 | .82 |
| T_4_ Life Satisfaction | 3.58 | .82 | 3.59 | .85 | 3.69 | .82 | 3.35 | .89 |
| T_1_ Elevation | 3.82 | 1.12 | 3.87 | 1.30 | 3.95 | 1.18 | 3.8 | 1.16 |
| T_2_ Elevation | 3.86 | 1.29 | 4.11 | 1.35 | 4.01 | 1.28 | 3.80 | 1.33 |
| T_3_ Elevation | 3.95 | 1.36 | 4.07 | 1.48 | 3.99 | 1.41 | 3.63 | 1.34 |
| T_4_ Elevation | 4.02 | 1.39 | 4.05 | 1.46 | 4.12 | 1.47 | 3.54 | 1.47 |
| T_1_ Connectedness | 3.78 | .80 | 3.87 | .83 | 3.96 | .81 | 3.83 | .85 |
| T_2_ Connectedness | 3.85 | .84 | 3.96 | .79 | 3.97 | .82 | 3.83 | .89 |
| T_3_ Connectedness | 3.98 | .83 | 4.08 | .80 | 4.09 | .80 | 3.86 | .87 |
| T_4_ Connectedness | 4.09 | .78 | 4.22 | .73 | 4.18 | .78 | 3.87 | .89 |
| T_1_ Support | 4.22 | .67 | 4.24 | .63 | 4.36 | .61 | 4.31 | .64 |
| T_2_ Support | 4.25 | .64 | 4.28 | .61 | 4.36 | .56 | 4.22 | .58 |
| T_3_ Support | 4.24 | .63 | 4.30 | .62 | 4.38 | .61 | 4.23 | .59 |
| T_4_ Support | 4.35 | .58 | 4.38 | .61 | 4.41 | .58 | 4.21 | .62 |
| T_1_ Loneliness | 2.95 | 1.18 | 2.93 | 1.19 | 2.83 | 1.15 | 2.90 | 1.16 |
| T_2_ Loneliness | 2.51 | .93 | 2.40 | .95 | 2.38 | .97 | 2.43 | .92 |
| T_3_ Loneliness | 2.37 | .95 | 2.29 | .98 | 2.26 | .94 | 2.31 | .91 |
| T_4_ Loneliness | 2.25 | .96 | 2.20 | .96 | 2.20 | .98 | 2.32 | .99 |

**Table S2**

*Regressed Change Models for Hypotheses 1 and 2*

|  |  |  |  |  |  | Partial *d* 95% CI | |
| --- | --- | --- | --- | --- | --- | --- | --- |
| Outcome | *b* | *b SE* | *t* | *p* | Partial *d* | LL | UL |
| **Hypothesis 1. Gratitude > Control:** | | | | | | | |
| Gratitude | .28 | .05 | 5.62 | <.001 | .38 | .25 | .51 |
| Positive Emotions | .35 | .09 | 3.96 | <.001 | .27 | .13 | .40 |
| Negative Emotions | -.07 | .07 | -0.99 | .321 | -.07 | -.20 | .07 |
| Social Emotions | -.02 | .05 | -0.33 | .742 | -.02 | -.15 | .11 |
| Life Satisfaction | .17 | .04 | 4.01 | <.001 | .27 | .14 | .40 |
| Elevation | .47 | .09 | 5.30 | <.001 | .36 | .23 | .49 |
| Connectedness | .28 | .05 | 5.58 | <.001 | .38 | .24 | .51 |
| Support | .19 | .04 | 5.24 | <.001 | .35 | .22 | .49 |
| Loneliness | -.11 | .05 | -2.25 | .025 | -.15 | -.28 | -.02 |
| **Hypothesis 2. 1-to1 > Others:** | | | | | | | |
| Gratitude | .07 | .05 | 1.33 | .184 | .09 | -.04 | .22 |
| Positive Emotions | .09 | .09 | 1.00 | .316 | .07 | -.06 | .20 |
| Negative Emotions | .04 | .07 | 0.51 | .609 | .03 | -.10 | .17 |
| Social Emotions | -.04 | .05 | -0.81 | .419 | -.05 | -.19 | .08 |
| Life Satisfaction | .01 | .04 | 0.19 | .852 | .01 | -.12 | .15 |
| Elevation | .12 | .09 | 1.38 | .166 | .09 | -.04 | .23 |
| Connectedness | .17 | .05 | 3.32 | .001 | .22 | .09 | .36 |
| Support | .09 | .04 | 2.32 | .020 | .16 | .02 | .29 |
| Loneliness | -.07 | .05 | -1.49 | .135 | -.10 | -.23 | .03 |

*Note.* Hypothesized condition dummy codes predicting T_4_ scores, controlling for T_1_ scores. For hypothesis 1, positive bs suggest the gratitude groups reported greater increases than the control group; while negative *b*s suggest the control group reported greater increases than the gratitude groups. For hypothesis 2, positive *b*s suggest the 1-to-1 group reported greater increases than the other groups; while negative *b*s suggest the other groups reported greater increases than the 1-to-1 group. CI = confidence interval; LL = lower limit; UL = upper limit.

**Table S3**

*Multilevel Model vs. Regressed Change Model Effect Sizes for Hypotheses 1 and 2*

| Outcome | Multilevel  Model  Partial *d* | Regressed  Change  Partial *d* |
| --- | --- | --- |
| **Hypothesis 1. Gratitude > Control:** | | |
| Gratitude | .36 | .38 |
| Positive Emotions | .29 | .27 |
| Negative Emotions | -.12 | -.07 |
| Social Emotions | -.02 | -.02 |
| Life Satisfaction | .17 | .27 |
| Elevation | .35 | .36 |
| Connectedness | .33 | .38 |
| Support | .32 | .35 |
| Loneliness | -.13 | -.15 |
| **Hypothesis 2. 1-to-1 > Others:** | | |
| Gratitude | .09 | .09 |
| Positive Emotions | .05 | .07 |
| Negative Emotions | .00 | .03 |
| Social Emotions | -.07 | -.05 |
| Life Satisfaction | -.04 | .01 |
| Elevation | .07 | .09 |
| Connectedness | .18 | .22 |
| Support | .16 | .16 |
| Loneliness | -.09 | -.10 |

**Table S4**

*Additional Condition Comparisons*

|  |  |  |  |  |  | Partial *d* 95% CI | |
| --- | --- | --- | --- | --- | --- | --- | --- |
| Outcome | *b* | *b SE* | *t* | *p* | Partial *d* | LL | UL |
| **Social vs. Nonsocial:** |  |  |  |  |  |  |  |
| Gratitude | -.03 | .05 | -0.55 | .585 | -.04 | -.20 | .11 |
| Positive Emotions | .00 | .09 | 0.00 | .996 | .00 | -.15 | .15 |
| Negative Emotions | .11 | .08 | 1.50 | .134 | .12 | -.04 | .27 |
| Social Emotions | -.08 | .05 | -1.64 | .101 | -.13 | -.28 | .03 |
| Life Satisfaction | -.01 | .05 | -0.29 | .773 | -.02 | -.18 | .13 |
| Elevation | .00 | .09 | -0.02 | .984 | .00 | -.16 | .15 |
| Connectedness | .05 | .05 | 0.89 | .371 | .07 | -.08 | .22 |
| Support | .00 | .04 | 0.06 | .952 | .00 | -.15 | .16 |
| Loneliness | -.01 | .05 | -0.11 | .915 | -.01 | -.16 | .15 |
| **Private vs. Others:** |  |  |  |  |  |  |  |
| Gratitude | .13 | .05 | 2.49 | .013 | .17 | .04 | .30 |
| Positive Emotions | .12 | .09 | 1.35 | .176 | .09 | -.04 | .22 |
| Negative Emotions | -.12 | .07 | -1.73 | .083 | -.12 | -.25 | .02 |
| Social Emotions | .06 | .05 | 1.34 | .181 | .09 | -.04 | .22 |
| Life Satisfaction | .07 | .04 | 1.68 | .094 | .11 | -.02 | .25 |
| Elevation | .17 | .09 | 1.84 | .066 | .12 | -.01 | .26 |
| Connectedness | .06 | .05 | 1.18 | .238 | .08 | -.05 | .21 |
| Support | .07 | .04 | 1.77 | .077 | .12 | -.01 | .25 |
| Loneliness | -.03 | .05 | -0.69 | .491 | -.05 | -.18 | .09 |
| **Public vs. Others:** |  |  |  |  |  |  |  |
| Gratitude | .10 | .05 | 1.86 | .063 | .13 | -.01 | .26 |
| Positive Emotions | .16 | .09 | 1.68 | .092 | .11 | -.02 | .25 |
| Negative Emotions | .02 | .07 | 0.22 | .823 | .02 | -.12 | .15 |
| Social Emotions | -.04 | .05 | -0.90 | .370 | -.06 | -.19 | .07 |
| Life Satisfaction | .10 | .05 | 2.25 | .025 | .15 | .02 | .28 |
| Elevation | .20 | .09 | 2.13 | .033 | .14 | .01 | .28 |
| Connectedness | .06 | .05 | 1.09 | .277 | .07 | -.06 | .21 |
| Support | .05 | .04 | 1.17 | .242 | .08 | -.05 | .21 |
| Loneliness | .00 | .05 | -0.08 | .939 | -.01 | -.14 | .13 |

Table S4 (continued)

|  |  |  |  |  |  | Partial *d* 95% CI | |
| --- | --- | --- | --- | --- | --- | --- | --- |
| Outcome | *b* | *b SE* | *t* | *p* | Partial *d* | LL | UL |
| **Private vs. Control:** |  |  |  |  |  |  |  |
| Gratitude | .30 | .06 | 4.91 | <.001 | .46 | .28 | .65 |
| Positive Emotions | .35 | .11 | 3.06 | .002 | .29 | .10 | .47 |
| Negative Emotions | -.14 | .09 | -1.61 | .108 | -.15 | -.34 | .03 |
| Social Emotions | .04 | .06 | 0.62 | .538 | .06 | -.13 | .24 |
| Life Satisfaction | .18 | .05 | 3.39 | .001 | .32 | .13 | .50 |
| Elevation | .47 | .11 | 4.23 | <.001 | .40 | .21 | .58 |
| Connectedness | .25 | .06 | 3.96 | <.001 | .37 | .19 | .56 |
| Support | .19 | .05 | 4.09 | <.001 | .38 | .20 | .57 |
| Loneliness | -.11 | .06 | -1.70 | <.001 | -.16 | -.34 | .03 |
| **1-to-1 vs. Control:** |  |  |  |  |  |  |  |
| Gratitude | .25 | .06 | 4.03 | <.001 | .38 | .19 | .56 |
| Positive Emotions | .33 | .11 | 2.97 | .003 | .28 | .09 | .46 |
| Negative Emotions | -.03 | .09 | -0.29 | .773 | -.03 | -.21 | .16 |
| Social Emotions | -.04 | .06 | -0.58 | .560 | -.05 | -.24 | .13 |
| Life Satisfaction | .13 | .05 | 2.48 | .013 | .23 | .05 | .42 |
| Elevation | .44 | .11 | 4.12 | <.001 | .39 | .20 | .57 |
| Connectedness | .33 | .06 | 5.50 | <.001 | .52 | .33 | .70 |
| Support | .21 | .04 | 4.79 | <.001 | .45 | .26 | .63 |
| Loneliness | -.14 | .06 | -2.34 | .020 | -.22 | -.40 | -.04 |
| **Public vs. Control:** |  |  |  |  |  |  |  |
| Gratitude | .28 | .06 | 4.40 | <.001 | .43 | .24 | .62 |
| Positive Emotions | .39 | .12 | 3.34 | .001 | .32 | .13 | .51 |
| Negative Emotions | -.04 | .09 | -0.45 | .656 | -.04 | -.23 | .15 |
| Social Emotions | -.04 | .06 | -0.71 | .475 | -.07 | -.26 | .12 |
| Life Satisfaction | .21 | .06 | 3.67 | <.001 | .36 | .17 | .55 |
| Elevation | .50 | .12 | 4.32 | <.001 | .42 | .23 | .61 |
| Connectedness | .25 | .07 | 3.71 | <.001 | .36 | .17 | .55 |
| Support | .17 | .05 | 3.82 | <.001 | .37 | .18 | .56 |
| Loneliness | -.08 | .06 | -1.32 | .187 | -.13 | -.32 | .06 |

*Note.* Additional condition comparisons conducted with regressed change models that used hypothesized condition dummy codes to predict T_4_ scores, controlling for T_1_ scores. First group received a “1,” and the second (reference) group received a “.” Social = 1-to-1 and Public gratitude conditions; Nonsocial = Private gratitude condition. Others = All other conditions. CI = confidence interval; LL = lower limit; UL = upper limit.

**Moderator Analyses**

Given the results of the additional condition comparisons described above, we conducted exploratory moderator analyses using the Hypothesis 1 dummy-coded pseudovariate that compared all three gratitude groups to the control condition. To reduce the number of comparisons, we conducted regressed change moderator analyses only for the outcome of life satisfaction, which is one of the most cited and well-used measures of subjective well-being in empirical literature (Diener et al, 2010). We predicted T_4_ scores from an interaction term (condition comparison dummy code * moderator), controlling for T_1_ scores for the following preregistered moderators: age, sex, ethnicity (Asian, Hispanic, White, and Black), subjective socioeconomic status (SES), extraversion (α =.69), neuroticism (α =.72), effort (“How much effort did you put into {the assigned activity}?”1 = *No effort*; 7 = *A great deal of effort*), participant diligence (e.g., “I carefully read every survey item”; 1 = *Strongly disagree*; 7 = *Strongly agree*; α =62), study duration (3-15 days). Although we also preregistered the personality traits of openness, conscientiousness, and agreeableness as exploratory moderator variables, the alphas for these variables (α =.37, .58, and .47, respectively) fell below the acceptable α =.60 threshold, so were removed from analyses. We also explored one additional moderator that was not preregistered, start month (a variable that assessed which month participants began the study), as data were collected just before and during the COVID-19 pandemic (February through December 2020). Overall, none of these moderator analyses were significant, suggesting that participants in all three gratitude groups did not benefit more (or less) depending on their age, gender, ethnicity, SES, personality, how much effort they put into the activity, how diligently they attended to the survey items, how long it took them to complete the study, or which month they started the study in 2020 (see Table S5).

**Table S5**

*Moderator Analyses*

|  |  |  | |  | |  | |  | | Partial d 95% CI | |
| --- | --- | --- | --- | --- | --- | --- | --- | --- | --- | --- | --- |
| Moderator | *b* | | *b SE* | | *t* | | *p* | | Partial *d* | LL | UL |
| Age | -.02 | | .02 | | -1.11 | | .266 | | -.08 | -.21 | .06 |
| Sex | .04 | | .10 | | 0.43 | | .665 | | .03 | -.10 | .16 |
| Asian | -.07 | | .09 | | -0.81 | | .419 | | -.05 | -.19 | .08 |
| Hispanic | .07 | | .09 | | 0.80 | | .425 | | .05 | -.08 | .19 |
| White | .18 | | .15 | | 1.22 | | .223 | | .08 | -.05 | .22 |
| Black | -.30 | | .22 | | -1.41 | | .159 | | -.10 | -.23 | .04 |
| SES | -.01 | | .03 | | -0.49 | | .622 | | -.03 | -.17 | .10 |
| Extraversion | -.01 | | .04 | | -0.20 | | .838 | | -.01 | -.15 | .12 |
| Neuroticism | .06 | | .04 | | 1.44 | | .152 | | .10 | -.04 | .23 |
| Effort | .02 | | .01 | | 1.72 | | .086 | | .12 | -.02 | .25 |
| Diligence | .00 | | .06 | | 0.04 | | .969 | | .00 | -.13 | .14 |
| Study Duration | .03 | | .03 | | 0.93 | | .351 | | .06 | -.07 | .20 |
| Start Month | -.01 | | .01 | | -0.60 | | .549 | | -.04 | -.17 | .09 |

*Note.* Outcome = Life Satisfaction. Condition comparison = Hypothesis 1: Gratitude vs. Control. To reduce the number of comparisons, we conducted regressed change moderator analyses for the outcome of life satisfaction only. We predicted T_4_ scores from an interaction term (condition comparison dummy code * moderator), controlling for T_1_ scores. For the condition comparison dummy code, all three gratitude groups received a “1” and the control group received a “0.” SES = Subjective socioeconomic status; CI = confidence interval; LL = lower limit; UL = upper limit.

**Mediator Analyses**

Given the results of the additional condition comparisons described above, as we did with moderator analyses, we conducted exploratory mediator analyses using only the Hypothesis 1 pseudovariate (comparing participants in all three gratitude groups to control) and the outcome of life satisfaction. We wanted to determine whether expressing gratitude (privately, one-to-one, and publicly) led to relatively greater life satisfaction via increases in social emotions, elevation, connectedness, support, and/or loneliness over time. Mediator analyses were conducted using Hayes’ Process (Version 4.1) for R with 95% confidence intervals and 5,000 bootstrapped samples. All continuous variables were standardized. We found that expressing gratitude predicted greater elevation throughout the study (a path: β = .37, *p* < .001), and elevation predicted greater life satisfaction at posttest (b path: β = .21, *p* < .001). Bootstrap confidence intervals indicated a significant indirect effect for elevation (estimate = .08, 95% CI [.04, .12]). Expressing gratitude also predicted greater connectedness (a path: β = .35, *p* < .001), and connectedness predicted greater life satisfaction (b path: β = .08, *p* = .01). Bootstrap confidence intervals also indicated a significant indirect effect for connectedness (estimate = .03, 95% CI [.01, .06]). The other tested mediators of social emotions, support, and loneliness did not show indirect effects above zero to a statistically significant degree (see Table S6 and Figure S2).

**Table S6**

*Mediator Analyses*

|  |  | Effect of IV on M (a) | |  | Effect of M on DV (b) | |  | Indirect effect (ab) | |  | Direct effect (c') | | |
| --- | --- | --- | --- | --- | --- | --- | --- | --- | --- | --- | --- | --- | --- |
| Mediator |  | β *(SE)* | *p* |  | β *(SE)* | *p* |  | Effect (Boot *SE*) | (LLCI, ULCI) |  | Effect (*SE*) | *p* | (LLCI, ULCI) |
| Social Emotions |  | -.03 (.08) | .734 |  | .03 (.03) | .305 |  | -.001 (.004) | (-.010, .008) |  | .31 (.08) | <.001 | (.16, .46) |
| Elevation |  | .37 (.08) | <.001 |  | .21 (.03) | <.001 |  | **.08 (.02)** | **(.04, .12)** |  | .23 (.08) | .002 | (.08, .38) |
| Connectedness |  | .35 (.08) | <.001 |  | .08 (.03) | .010 |  | **.03 (.02)** | **(.01, .06)** |  | .28 (.08) | <.001 | (.13, .43) |
| Support |  | .37 (.08) | <.001 |  | .04 (.03) | .195 |  | .02 (.02) | (-.01, .05) |  | .29 (.08) | <.001 | (.14, .44) |
| Loneliness |  | -.14 (.08) | .064 |  | .04 (.03) | .207 |  | -.01 (.01) | (-.02, .004) |  | .32 (.08) | <.001 | (.16, .46) |

*Note.* Mediation analyses conducted using Hayes’ Process for R (version 4.1) with 95% confidence intervals and 5,000 bootstrapped samples. All continuous variables were standardized. Total effect (c) was β = .31 (*SE* = .08), *p* <.001, (LLCI=.16, ULCI=.46) for all mediators. IV = independent variable (dummy coded condition comparison of hypothesis 1: gratitude vs. control); DV = dependent variable/outcome (life satisfaction); CI = confidence interval; LL = lower limit; UL = upper limit. **Bold** indirect effects indicate positive effects above zero to a statistically significant degree.

**Figure S2**

*The Effect of Gratitude on Life Satisfaction is Mediated by Elevation and Connectedness*


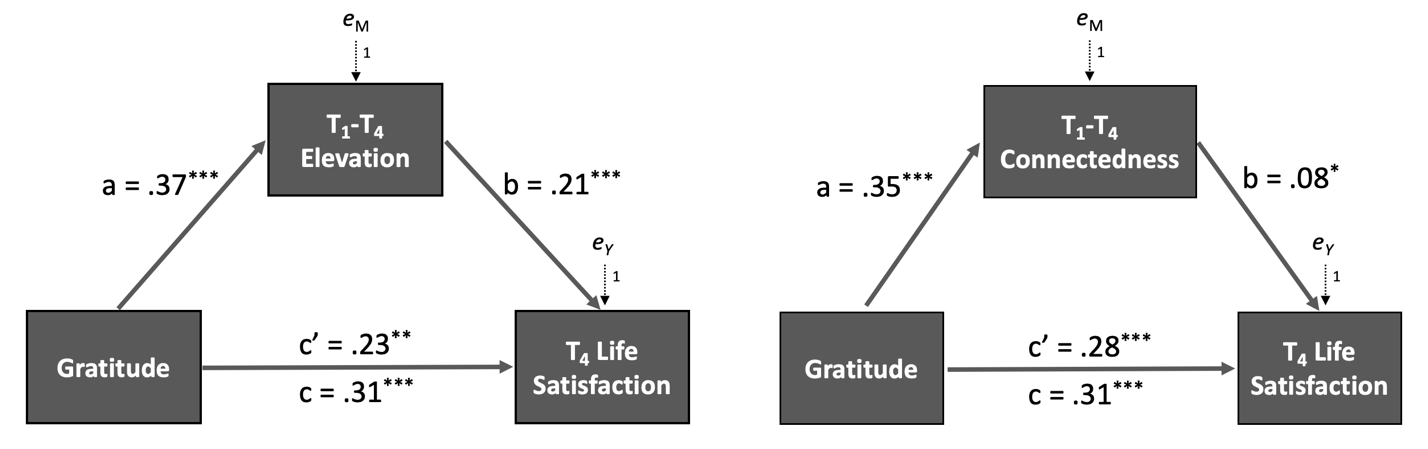


Note. The effect of expressing gratitude on life satisfaction via increased elevation (left) and connectedness (right). All continuous variables were standardized. *p < .05. **p < .01.
***p < .001.

**Retrospective Change Analyses**

Finally, we preregistered and ran regressed change analyses for each of three retrospective change assessments. These assessments asked participants to rate their level of agreement on a few statements (“I am happy,” “I am grateful,” and “I feel connected to others”) to indicate how much they believed they changed over the course of the study (Little et al., 2019). Participants did this retrospectively, on two 100-point sliders asking them to indicate their level of happiness “Before this study” and “At this time.” Using our preregistered dummy coded pseudovariates for Hypotheses 1 and 2, we predicted “At this time” scores from condition pseudovariates, controlling for “Before this study” scores. Relative to control, those in the gratitude groups reported feeling happier (*b* = 8.25, *p* < .001, partial *d* = .48), more grateful (*b* = 8.51, *p* < .001, partial *d* = .58), and more connected (*b* = 8.23, *p* < .001, partial *d* = .45) at the end of the study (see Table S7 Hypothesis 1. Gratitude > Control). Relative to all other groups, those in the *Gratitude 1-to-1* group reported feeling happier (*b* = 4.22, *p* < .001, partial *d* = .24) and more connected (*b* = 3.32, *p* = .009, partial *d* = .18), but not more grateful (see Table S7 Hypothesis 2. 1-to1 > Others).

**Table S7**

*Retrospective Change Analyses*

|  |  |  |  |  |  | Partial *d* | |
| --- | --- | --- | --- | --- | --- | --- | --- |
| Outcome | *b* | *b SE* | *t* | *p* | Partial *d* | LL | UL |
| **Hypothesis 1. Gratitude > Control:** | |  |  |  |  |  |  |
| Happy | 8.25 | 1.17 | 7.06 | <.001 | .48 | .35 | .61 |
| Grateful | 8.51 | 0.99 | 8.61 | <.001 | .58 | .45 | .71 |
| Connected | 8.23 | 1.24 | 6.63 | <.001 | .45 | .32 | .58 |
| **Hypothesis 2. 1-to-1 > Others:** | |  |  |  |  |  |  |
| Happy | 4.22 | 1.19 | 3.53 | <.001 | .24 | .11 | .37 |
| Grateful | 1.39 | 1.03 | 1.35 | .177 | .09 | -.04 | .22 |
| Connected | 3.32 | 1.27 | 2.62 | .009 | .18 | .04 | .31 |

*Note.* Retrospective change conducted with regressed change models that used hypothesized condition dummy codes to predict “After” scores, controlling for “Before” scores.

CI = confidence interval; LL = lower limit; UL = upper limit.

**Condition Instructions**

**Private Gratitude Condition**

In our daily lives, we often thank people for both large and small things. However, it can sometimes be difficult to make time to express our gratitude more deeply. **Today, we want you to write a letter of gratitude to someone who has done something for which you are extremely grateful. Please do not share your letter with this person or anyone else.** Think of the people – partners, parents, siblings, relatives, friends, neighbors, mentors, and so on – who have been especially generous and thoughtful towards you. For example, you may feel grateful to a parent who has always been there for you, or to a friend who helped you through a hard time. Now we would like you to pick ONE person who helped you, and write a letter of gratitude to them. However, this letter is really for you to express your thoughts—it is not to convey your gratitude directly to the other person. In fact, **we do not want you to share your letter with anyone at all.** You could type this letter out on your computer or start a note on your smartphone. To give you some ideas of what to write, you could describe the kind act(s) this person did for you and how the kind act(s) affected your life. You could also describe what you are doing now and how you often remember their efforts. We will send you a follow-up survey tomorrow to ask how it went.

**1-to-1 Gratitude Condition**

In our daily lives, we often thank people for both large and small things. However, it can sometimes be difficult to make time to express our gratitude more deeply. **Today, we want you to use your smartphone to text someone who has done something for which you are extremely grateful, and thank them for their kind act(s).** Think of the people – partners, parents, siblings, relatives, friends, neighbors, mentors, and so on – who have been especially generous and thoughtful towards you. For example, you may feel grateful to a parent who has always been there for you, or to a friend who helped you through a hard time.   Now we would like you to pick ONE person who helped you, and text them to share your gratitude. **Please text ONLY the person who helped you directly via a private message.** That is, we do not want you to text that person in a group message where other people can see it. To give you some ideas of what to say, you could describe the kind act(s) this person did for you and how the kind act(s) affected your life. You could also describe what you are doing now and how you often remember their efforts. We will send you a follow-up survey tomorrow to ask how it went.

**Public Gratitude Condition**

In our daily lives, we often thank people for both large and small things. However, it can sometimes be difficult to make time to express our gratitude more deeply. **Today, we want you to use social media to reach out to someone who has done something for which you are extremely grateful, and thank them for their kind act(s).**  Think of the people – partners, parents, siblings, relatives, friends, neighbors, mentors, and so on – who have been especially generous and thoughtful towards you. For example, you may feel grateful to a parent who has always been there for you, or to a friend who helped you through a hard time.  Now we would like you to pick ONE person who helped you, and share your gratitude with that person on social media. That is, we would like you to publicly post/tweet/share your gratitude with this person and tag/mention that person. You can use any social media platform you like (e.g., Instagram, Facebook, Twitter). **We just ask that you thank this person publicly (in a post/tweet; not in a private message).** To give you some ideas of what to say, you could describe the kind act(s) this person did for you and how the kind act(s) affected your life. You could also describe what you are doing now and how you often remember their efforts. We will send you a follow-up survey tomorrow to ask how it went.

**Control Condition**

Our daily lives are filled with different activities, and it can sometimes be challenging to organize our time.  **Today, we want you to keep track of all the activities you do.** Examples of activities include things like going to work, exercising at the gym, watching TV, and eating a meal. Today, we would like you to track your activities. To do this, you could type your activities out on your computer or start a note on your smartphone to keep a brief log of everything you do.  There is no need to remember who you are with or how you are feeling. Instead, just try to remember factual information about what you are doing.  Do not alter your routine in any way; simply keep track of what you do. We will send you a follow-up survey tomorrow to ask how it went.
